# Food for all? Wildfire ash fuels growth of diverse eukaryotic plankton

**DOI:** 10.1098/rspb.2023.1817

**Published:** 2023-11-01

**Authors:** T. M. Ladd, D. Catlett, M. A. Maniscalco, S. M. Kim, R. L. Kelly, S. G. John, C. A. Carlson, M. D. Iglesias-Rodríguez

**Affiliations:** ^1^ Interdepartmental Graduate Program in Marine Science, University of California, Santa Barbara, CA, USA; ^2^ Marine Science Institute, University of California, Santa Barbara, CA, USA; ^3^ Department of Ecology, Evolution, and Marine Biology, University of California, Santa Barbara, CA, USA; ^4^ Department of Earth Sciences, University of Southern California, Los Angeles, CA, USA

**Keywords:** marine microbial communities, California wildfires, nutrient fertilization, protists, wildfire ash

## Abstract

In December 2017, one of the largest wildfires in California history, the Thomas Fire, created a large smoke and ash plume that extended over the northeastern Pacific Ocean. Here, we explore the impact of Thomas Fire ash deposition on seawater chemistry and the growth and composition of natural microbial communities. Experiments conducted in coastal California waters during the Thomas Fire revealed that leaching of ash in seawater resulted in significant additions of dissolved nutrients including inorganic nitrogen (nitrate, nitrite and ammonium), silicic acid, metals (iron, nickel, cobalt and copper), organic nitrogen and organic carbon. After exposure to ash leachate at high (0.25 g ash l^−1^) and low (0.08 g ash l^−1^) concentrations for 4 days, natural microbial communities had 59–154% higher particulate organic carbon concentrations than communities without ash leachate additions. Additionally, a diverse assemblage of eukaryotic microbes (protists) responded to the ash leachate with taxa from 11 different taxonomic divisions increasing in relative abundance compared with control treatments. Our results suggest that large fire events can be important atmospheric sources of nutrients (particularly nitrogen) to coastal marine systems, where, through leaching of various nutrients, ash may act as a ‘food for all’ in protist communities.

## 1. Introduction

**Figure 1.**
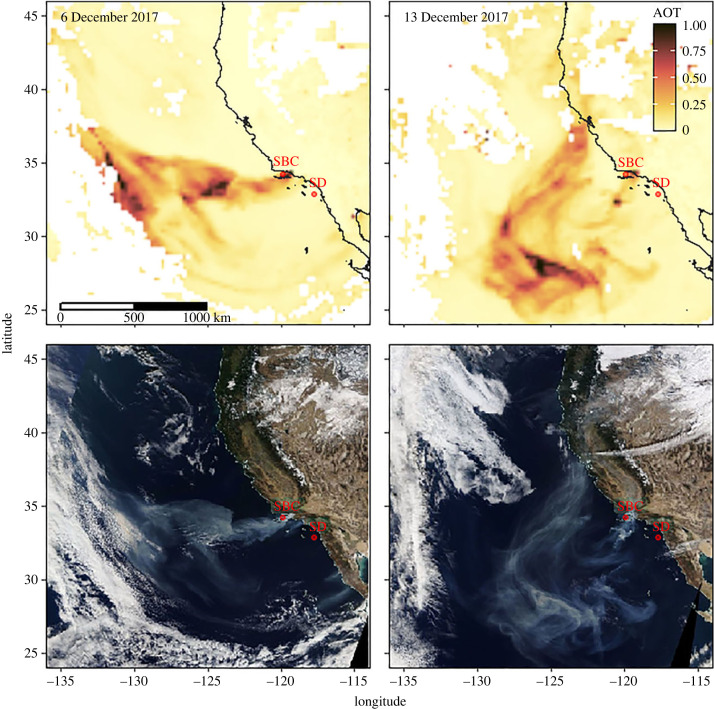
Thomas Fire smoke and ash plume. Aerosol optical thickness (AOT) gridded data (SNPP/VIIRS) and true colour imagery (MODIS Terra) of the Thomas Fire plume on 6 and 13 December 2017. The two incubation water collection locations are indicated as red circles with SD and SBC located outside and within the plume, respectively.

Worldwide, many regions are experiencing changing wildfire activity due to both climatic and anthropogenic forcings [1–3]. Recent occurrences of large wildfires in many locations around the world and their apparent increasing frequency demonstrate the need to better understand the impacts of these events on both local and global biogeochemical processes [1]. In California specifically, observations and modelling studies show that fires will continue to increase in frequency and severity as the climate changes and humans continue to live near and encroach on fire-prone ecosystems [4–6]. In December 2017, the Thomas Fire became the largest California fire in modern history, though it has since been surpassed by seven other fires [7]. The Thomas Fire ignited in Ventura County (CA, USA) on 4 December 2017, and burned approximately 1140 km^2^ of coastal terrain dominated by chapparal and oak woodland as well as over 1000 structures before being fully contained on 12 January 2018 [8,9]. Satellite imagery showed a large plume of smoke and ash that extended more than 1000 km offshore near the Santa Barbara Channel (SBC) and led to persistent but spatially variable ash deposition into the SBC throughout December (figure 1). Although atmospheric aerosols from sources such as dust, volcanos and anthropogenic pollution are known to provide a variety of chemical compounds to oceanic systems that significantly alter marine biogeochemical cycles, wildfire sources have rarely been considered.

Wildfire ash is a complex chemical mixture of particulate material remaining after the burning of wildland fuels that consists of both minerals and charred organic components [10]. California wildfires have been shown to alter the chemistry of freshwater runoff and streams by increasing pH and the concentrations of nutrients (e.g. nitrate, ammonium and phosphate), metals and hydrocarbons [11–13]. Owing to the complex chemistry of ash and variability in composition based on fire conditions and fuel sources, it is difficult to predict if dissolution in seawater will result in fertilizing or toxic effects and how these effects might vary based on ash concentration and biological community composition. In a limited number of studies on marine systems, wildfires have been suggested to supply critical nutrients required for primary production with observed increases in atmospheric deposition of metals [14–16] or macronutrients (e.g. nitrogen and phosphorus) [17] in wildfire-adjacent coastal marine systems. Additionally, a study on the 2019–2020 Australian wildfires concluded that increased iron concentrations due to deposition of wildfire aerosols led to anomalously high chlorophyll concentrations thousands of kilometres away in the Southern Ocean [16]. In freshwater systems, several studies have demonstrated that wildfire ash and post-fire runoff can be toxic to a variety of organisms across multiple trophic levels [18–22]. Toxic effects on some marine microbes have also been observed during exposure to dust or volcanic aerosols presumably due to high copper concentrations [23,24]. While these studies have suggested that phytoplankton productivity may be enhanced by nutrient deposition from wildfires [15–17,25] or that aerosols or wildfire ash can be toxic to organisms [18–24], the mechanisms and short-term responses of natural microbial communities remains largely unknown.

**Figure 2.**
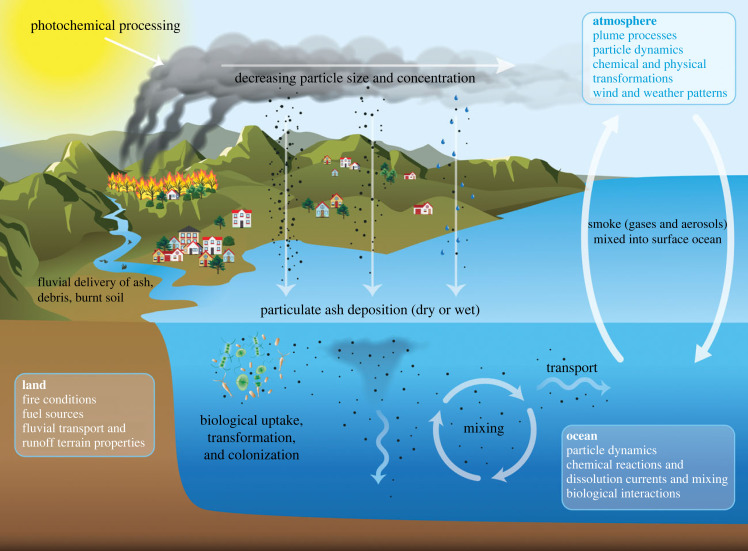
Conceptual diagram illustrating potential mechanisms and processes impacting delivery of wildfire-derived materials to marine systems.

Here, we used wildfire ash derived from the Thomas Fire and natural microbial communities collected from coastal California waters (figure 1) to explore the impacts of wildfire ash on seawater chemistry and single-celled eukaryotic plankton (protist) community composition. We focus on dry deposition of wildfire ash as a major driver of biogeochemical changes in the marine environment, but we acknowledge that there are various additional mechanisms for wildfires to affect marine systems that depend on combined processes on land, in the atmosphere and in the ocean (figure 2). Through short-term experimental incubations, we show that dry deposition of wildfire ash has the potential to fertilize coastal marine microbial communities by leaching a diverse mixture of inorganic nitrogen species and other nutrients when deposited in seawater. Additionally, our findings suggest that wildfire ash addition results in the growth of a diverse assemblage of marine protists rather than only a few opportunistic species. Thus, we conclude that wildfire ash can be an important source of nutrients, particularly nitrogen, to coastal ecosystems where we hypothesize that ash serves as a ‘food for all’ in marine protist communities by supplying various types of nutrients in small pulses.

## 2. Material and methods

### (a) Environmental setting and experimental setup

Thomas Fire ash was collected at several locations near actively burning areas. Ash leachate was created by mixing 20 g of bulk, homogenized Thomas Fire ash to 1 l of filtered seawater (20 g ash l^−1^) and subsequently filtering to remove particulate material (electronic supplementary material, Materials and Methods). Incubation experiments exposing natural microbial communities to wildfire ash leachate were conducted on the RV *Sally Ride* from 16 to 21 December 2017 using seawater collected offshore of San Diego, California (SD) (32.867°, −117.734°) and seawater from within the SBC (34.250°, −119.906°) (figure 1). To create the experimental treatments, ash leachate was added to bottles containing 200 µm prefiltered SD or SBC seawater to obtain high ash (H) concentrations (0.25 g ash l^−1^), low ash (L) concentrations (0.08 g ash l^−1^) or no ash controls (C). Each experiment was conducted deckboard in replicate approximately 2.3 l polycarbonate bottles over 4 days with samples for particulate organic carbon (POC) concentrations (representing approximate community biomass), nutrient (nitrate, nitrite, phosphate, silicic acid) concentrations and single-celled eukaryotic plankton (protist) community composition (18S rDNA metabarcoding) collected at the start of each experiment and on days 2 and 4.

### (b) Sampling and analysis

The ash leachate used for the experiments conducted here plus additional ash leachate created by mixing Thomas Fire ash with filtered SBC seawater (electronic supplementary material, Materials and Methods) was used to analyse chemical changes in seawater due to ash addition. Aliquots of ash leachate were stored at −20°C until analysis for concentrations of inorganic nutrients (nitrate + nitrite, nitrite, ammonium, phosphate and silicic acid), total dissolved nitrogen (TDN) and phosphorus (TDP), dissolved organic carbon (DOC) and trace metals (Fe, Zn, Pb, Ni, Cd, Mn, Co and Cu) (electronic supplementary material, Materials and Methods). Changes in concentration due to ash addition were analysed by subtracting concentrations in the background seawater from the ash leachate concentrations.

Triplicate incubation bottles were sampled sacrificially at each time point for POC concentrations, inorganic nutrient concentrations (nitrate + nitrite, nitrite, phosphate and silicic acid) and 18S DNA metabarcoding. Samples for POC (500 ml) were vacuum filtered onto pre-combusted glass fibre filters (0.7 µm, 25 mm, Whatman) and stored in pre-combusted glass scintillation vials at −20°C. Acidified POC filters were analysed using the Dumas combustion method in an automated elemental analyser (model CE-440HA, Exeter Analytical) at the University of California, Santa Barbara (UCSB) Marine Science Institute (MSI) Analytical Laboratory. Inorganic nutrient samples (approx. 15 ml) were filtered through a 0.2 µm polycarbonate filter (Isopore, EMD-Millipore) into sample-rinsed plastic scintillation vials and frozen at −20°C until analysis. Inorganic nutrients were analysed by flow injection analysis (QuikChem 8000, Zellweger Analytics) at the UCSB MSI Analytical Laboratory. Samples for DNA metabarcoding (1–2 l) were vacuum filtered onto 1.2 µm polycarbonate filters (Isopore, EMD-Millipore), placed in 4 ml cryovials, and submerged in liquid nitrogen less than 15 min after collection. Samples were stored at −80°C until DNA extraction. Extraction of DNA from the filters followed the AllPrep Mini Kit (Qiagen) protocol after lysis of cells by bead beating in lysis buffer. Amplification of the V9 hypervariable region of the 18S rRNA gene and Illumina library preparation followed protocols described by Catlett *et al*. [26] (electronic supplementary material, Materials and Methods). Library sequencing was performed using a MiSeq PE150 v2 kit (Illumina) at the DNA Technologies Core of the University of California Davis Genome Center. Demultiplexed sequencing data were processed with the DADA2 pipeline (v1.16.0) [27] and followed procedures described in Catlett *et al*. [26] (electronic supplementary material, Materials and Methods). Before analysis, non-protistan amplicon sequence variants (ASVs) and ASVs that only appeared in a single sample were removed and sequences were subsampled without replacement to the minimum library size (15 818 reads per sample).

### (c) Statistical analysis

Statistical analyses were conducted using R (v4.0.1) and JMP Pro 15. Statistically significant additions (*p* < 0.05) of inorganic nutrients in ash leachate samples were determined using *t*-tests or Alexander–Govern tests after testing for normality of residuals (Shapiro–Wilk test) and homogeneity of variances (Levene test). Statistically significant additions (*p* < 0.05) of organic nutrients and metals in the leachate samples were determined using a paired *t*-test (or paired Wilcoxon signed-rank test) after analysing the distributions for normality. To determine differences in POC or nutrient concentrations between treatments at each time point or across incubations, data were analysed for normality of residuals and homogeneity of variances before conducting analysis of variance (ANOVA) tests and Tukey HSD post-hoc pairwise comparisons when necessary. For nutrient concentrations measured as below the analytical limit of detection (LOD) (nitrate + nitrite: 0.2 µM, nitrite: 0.1 µM, phosphate: 0.1 µM, ammonium: 0.2 µM, silicic acid 1.0 µM), values were replaced with ½ LOD and only used in statistical analyses when just 1 replicate was below the LOD.

Analysis of protist communities and sequence relative abundances (RAs) of taxonomic and trophic groups were conducted in R (v4.0.1) using the Phyloseq (v1.32.0), Vegan (v2.5.6), pairwiseAdonis (v0.0.1) and DESeq2 (v1.28.1) packages. Significant differences (*p*_adjusted_ < 0.05) in major taxonomic groups between treatments at days 2 and 4 were initially tested with one-way ANOVAs and followed up with pairwise *t*-tests for individual treatments (either H or L compared with C) for taxonomic groups that significantly differed (ANOVA *p* < 0.05) and *p*-values were adjusted for multiple testing across all tests at a given time point according to the Benjamini–Hochberg method. Differential RA of individual ASVs (prevalence > 1, maximum RA > 0.01%) in ash treatments (H or L) compared with the C treatment across both days 2 and 4 were analysed separately for each incubation with DESeq2 (*α* = 0.05).

## 3. Results

### (a) Seawater chemistry and nutrient leaching from Thomas Fire ash

**Figure 3.**
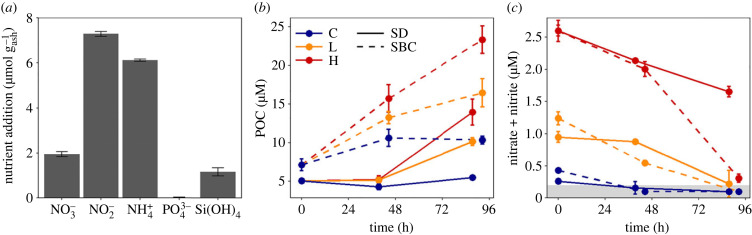
Nutrient and biomass dynamics in response to ash leaching. (*a*) Mean ± s.d. additions of nitrate (${\mathrm{NO}}_{3}^{-}$), nitrite (${\mathrm{NO}}_{2}^{-}$), ammonium (${\mathrm{NH}}_{4}^{+}$), phosphate (${\mathrm{PO}}_{4}^{3-}$) and silicic acid (Si(OH)_4_) (µmol g_(ash)_^−1^). (*b*) Mean ± s.d. (*n* = 3 except *n* = 2 for SD H) concentrations of POC over time in ash and control treatments of the SD and SBC incubations. (*c*) Mean ± s.d. concentrations of nitrate + nitrite over time in ash and control treatments of the SD and SBC incubations. Measurements are from triplicate bottles (*n* = 3), except at time 0 where values are from a single sample (C) or the average of a single sample and estimated concentrations from the leachate (ash treatments) (*n* = 2). Values in the shaded area represent measurements below the analytical limit of detection (0.2 µM).

Addition of ash to seawater caused leaching of major inorganic nutrients (figure 3*a*; electronic supplementary material, table S1). Thomas Fire ash addition significantly increased concentrations of nitrite ($7.3\pm 0.1\;\mu \mathrm{mol}\;{\mathrm{NO}}_{2}^{-}\;g_{\left(\mathrm{ash}\right)}^{-1}$), ammonium ($6.1\pm 0.2\;\mu \mathrm{mol}\;{\mathrm{NH}}_{4}^{+}\;g_{\left(\mathrm{ash}\right)}^{-1}$), nitrate (1.9± $0.1\;\mu \mathrm{mol}\;{\mathrm{NO}}_{3}^{+}\;g_{\left(\mathrm{ash}\right)}^{-1}$) and silicic acid (1.2 ± 0.2 µmol Si(OH)_4_ g_(ash)_^−1^) (*t*-tests or Alexander–Govern tests *p* < 0.01). However, phosphate concentrations ($0.01\pm 0.03\;{\mathrm{PO}}_{4}^{3-}\;g_{\left(\mathrm{ash}\right)}^{-1}$) were not significantly increased (*t*-test *p* = 0.328). Subsequent leaching tests using Thomas Fire ash also revealed increased concentrations of DON, DOC and metals (iron, nickel, cobalt and copper) following leaching in seawater (electronic supplementary material, tables S2 and S3).

At the time of the Thomas Fire, surface seawater in the area was relatively low in macronutrient concentrations (electronic supplementary material, table S4) with apparent deficiencies in nitrogen relative to phosphorus or iron (N : P < 16 : 1 mol N : mol P and N : Fe < 2133 : 1 mol N : mol Fe) [28]. Initial ratios of nitrate + nitrite to phosphate in the control surface seawater from SD and the SBC were approximately 1 : 1 and 1.5 : 1 mol N : mol P, respectively (electronic supplementary material, table S4) while in samples taken on 14 December during another study in the SBC [15], ratios of nitrate + nitrite to iron were found to range from approximately 24 : 1 to 500 : 1 mol N : mol Fe. By contrast, the Thomas Fire ash leachate was nitrogen-rich compared with phosphorus and iron (N : P > 16 : 1 mol N : mol P and N : Fe > 2133 : 1 mol N : mol Fe) [28]. The ratio of TDN to TDP added to seawater from ash leaching was approximately 240 : 1 mol N : mol P with non-significant additions of TDP and phosphate (figure 3*a*; electronic supplementary material, tables S1 and S2). Additions of dissolved inorganic nitrogen (nitrate + nitrite + ammonium) equal to approximately 15 µmol g_(ash)_^−1^ compared with iron additions of 0.3 ± 0.1 nmol g_(ash)_^−1^ resulted in N : Fe of approximately 50 000 : 1 mol N : mol Fe due to ash leaching (figure 3*a*; electronic supplementary material, tables S1–S3).

### (b) Particulate organic carbon and nutrient trends in experimental incubations

All incubations with natural microbial communities exhibited increased POC concentrations as nitrate + nitrite and phosphate concentrations decreased over time, but POC concentrations increased more in communities exposed to Thomas Fire ash leachate (figure 3*b,c*; electronic supplementary material, figure S1). Over the first 2 days of the incubations, the increase in POC in ash treatments relative to the control was more than twofold higher in the SBC compared with the SD incubations (H: *t*-test *p* = 0.02, L: *t*-test *p* = 0.02), but between days 2 and 4 increases in POC relative to controls were not significantly different between the SD and SBC incubations in either the H (*t*-test *p* = 0.4) or L (*t*-test *p* = 0.5) treatments, revealing a lagged growth response of the SD microbial communities to ash leachate. By the end of the incubations (day 4), POC in the SD and SBC incubations were 154% and 126% greater (H) and 84.5% and 59% greater (L) compared with the controls, respectively (Tukey HSD *p* < 0.05) (figure 3*b*). Initial concentrations of nitrate + nitrite in the H and L treatments were increased by approximately 2.0–2.4 µM and 0.6–0.9 µM, respectively, compared with controls, while phosphate concentrations were similar across treatments (figure 3*c*; electronic supplementary material, figure S1). By day 4, the concentrations of nitrate + nitrite and phosphate in ash-leachate-amended treatments were depleted (concentrations decreased by approx. 0.6–2 µM and approx. 0.1–0.2 µM, respectively) and oftentimes were below detection limits (figure 3*c*; electronic supplementary material, figure S1). By contrast, C treatments had very low initial nitrate + nitrite concentrations (approx. 0.2–0.4 µM) and were depleted to below detection limits (less than 0.2 µM) by day 2 while phosphate concentrations decreased much less than in the ash-leachate-amended treatments (decreases of only approx. 0.03–0.05 µM) (figure 3*c*; electronic supplementary material, figure S1).

### (c) Protist community response to ash

**Figure 4.**
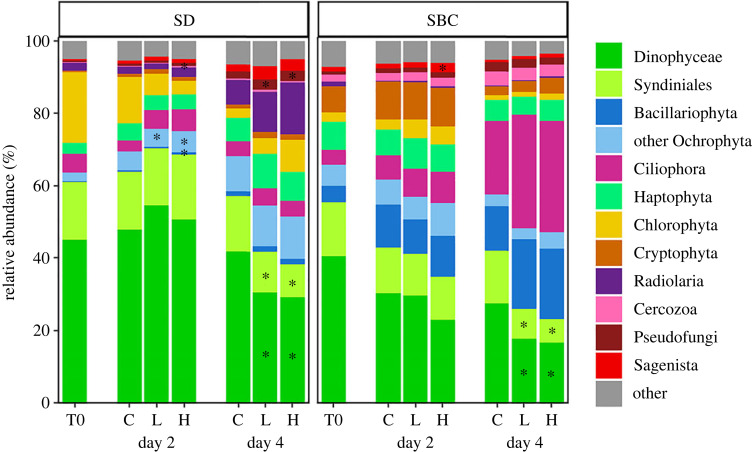
Protist community composition response to ash exposure. Mean RA of ASVs grouped by division or class (*n* = 3) in the SD and SBC incubation experiments at the start of each experiment (T0) and at days 2 and 4 for each treatment. Division Dinoflagellata has been divided to show the RA of classes Dinophyceae and Syndiniales, and division Ochrophyta was split into Bacillariophyta and other Ochrophyta classes. The ‘other’ group represents ASVs from less abundant divisions/classes (Telonemia, Katablepharidophyta, Discoba, Opalozoa, Picozoa, Apicomplexa, Choanoflagellida, Centroheliozoa, unassigned Alveolata, unassigned Stramenopiles, Mesomycetozoa, Lobosa, unassigned Amoebozoa, Foraminifera, Conosa and other Dinoflagellata classes). Asterisks represent divisions/classes with significantly different RA (pairwise *t*-tests, *p*_adj_ < 0.05) between either the H or L treatments compared with C on day 2 or day 4.

**Figure 5.**
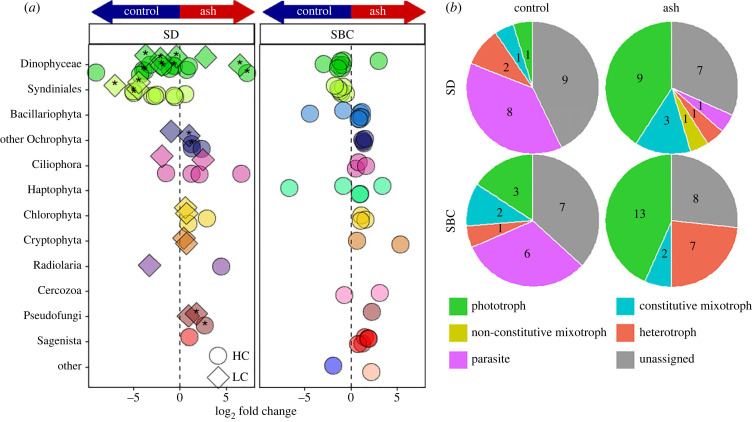
Significant responses of protist ASVs to ash exposure. (*a*) ASVs that were significantly differentially abundant (DESeq2, *p* < 0.05) between ash and control treatments in the SD and SBC incubations. Colours represent each ASV's division/class (denoted on the *y*-axis and coloured as in figure 4) and the shape of the point represents differentially abundant ASVs in either the H (circles) or L (diamonds) treatment relative to C. A negative (positive) log_2_ fold change indicates that an ASV is relatively more abundant in the control (ash) treatment(s). Asterisks represent ASVs that were significantly differentially abundant in both H and L treatments. ASVs that are classified as ‘other’ come from divisions Telonemia (violet) and Opalozoa (light pink). (*b*) The number of significantly differentially abundant ASVs as determined from DESeq2 (*p* < 0.05) coloured by their respective putative trophic strategies. The different pie charts represent ASVs with significantly higher RA in the control or ash treatments for the SD and SBC incubations.

Amplicon sequencing of the 18S-V9 rRNA gene revealed significant changes in protist community composition after exposure to Thomas Fire ash leachate (figures 4 and 5; electronic supplementary material, figures S2 and S3). The initial composition of the SD and SBC protist communities differed resulting in distinct changes in the communities in response to ash addition, although we also observed several commonalities in the responses of protist groups. When considering POC increases in conjunction with these results, the addition of ash appeared to enhance the growth of many members of the protist community simultaneously (figures 4 and 5; electronic supplementary material, figure S4).

In the SD incubation, ASVs from divisions Ochrophyta (class Bacilliaryophyta and other Ochrophyta), Cercozoa and Psuedofungi had significantly higher RA in the L or H treatments compared with controls on either day 2 or day 4 (pairwise *t*-tests, *p*_adjusted_ < 0.05), but not consistently across days (figure 4). In the SBC incubation, only a single division (Sagenista) displayed significantly higher RA in the H treatment compared with C on day 2 (pairwise *t*-test, *p*_adjusted_ = 0.0005) (figure 4). Across both incubation experiments the only consistent responses were reduced RAs of Dinophyceae and Syndiniales (division Dinoflagellata) ASVs in ash exposed treatments. After 4 days of ash exposure, the cumulative RA of Dinophyceae and Syndiniales ASVs in ash treatments was 9–13% and 4–8% lower than in controls, respectively (pairwise *t*-tests, *p*_adjusted_ < 0.05) (figure 4). Additionally, 77% (SD) and 93% (SBC) of significantly differentially abundant Dinoflagellata ASVs had lower RA in ash than in control treatments (figure 5*a*; electronic supplementary material, figure S3). To investigate whether the reduction in dinoflagellate RA was driven by a decrease in dinoflagellate biomass or less pronounced growth relative to other protists, we scaled protistan RA to concurrent estimates of POC concentrations (see electronic supplementary material, Materials and Methods). Despite the reduction in dinoflagellate RA, estimated Dinophyceae and Syndiniales POC increased slightly or was not significantly different in ash treatments compared with controls on day 4 (pairwise *t*-tests, *p*_adjusted_ < 0.05) (electronic supplementary material, figure S4).

Differential abundance testing of individual ASVs revealed 22 ASVs in the SD incubation and 30 ASVs in the SBC incubation from a total of 11 different taxonomic divisions that were determined to have significantly higher RAs in at least one ash treatment compared with controls across both incubations (figure 5*a*). Classification of ASVs to putative trophic strategies revealed that across both the SD and SBC incubations, ASVs with significantly higher RA in ash treatments were more commonly photoautotrophic (SD = 41%, SBC = 43%) than ASVs with higher RA in control treatments (SD = 5%, SBC = 16%) (figure 5*b*). Further, several of the ash-associated phototroph ASVs were identified as putative pico- or nano-phytoplankton (electronic supplementary material, table S5 and dataset S1).

## 4. Discussion

### (a) Nutrient fertilization via wildfire ash deposition and leaching

The enrichment of inorganic and organic nutrients in the Thomas Fire ash leachate was expected based on observations of wildfire ash in freshwater systems [11–13,29–31] and deposition of other atmospheric aerosols to marine systems [32,33]. Although the chemical composition of wildfire ash is known to vary based on factors including fire conditions, fuel type and atmospheric processes [10,22,34,35], our results from the Thomas Fire suggest that ash may be an important source of nitrogen and other inorganic and organic compounds that likely impact microbial community production and composition. Harper *et al*. [22] compared wildfire ash leachates (in deionized water) derived from six different fires around the world and discovered the leachate chemistry was hugely variable between sites [22]. Nitrate and phosphate additions in the different leachates ranged from 0.4 to 3.7 µmol g_(ash)_^−1^ and 0.01–6.5 µmol g_(ash)_^−1^, respectively, and the leachates were not always nitrogen-rich relative to phosphorus and iron [22]. Additionally, the ash leachate chemistry results reported here differed from the Thomas Fire ash leachate reported by Harper *et al*. [22] which leached higher amounts of nitrate (3.7 versus 1.9 µmol g_(ash)_^−1^), phosphate (0.1 versus 0.01 µmol g_(ash)_^−1^) and iron (0.01 versus 0.0003 µmol g_(ash)_^−1^) compared with our results but was still nitrogen-rich relative to phosphorus, although not compared with iron (although nitrite concentrations were not measured) [22].

The lack of phosphate and organic phosphorus leached from Thomas Fire ash was surprising given that wildfires have been shown to increase concentrations of atmospheric phosphorus [36] and dissolved phosphorus in fire impacted watersheds [12,37]. Although ash may contain significant quantities of phosphorus containing compounds, phosphorus in wildfire ash has low aqueous solubility [34,38–40] and the solubility of phosphorus is lower in seawater compared with freshwater [41,42]. Measurements during other fires have suggested that low intensity fires may produce less atmospheric phosphorus [43] while laboratory tests demonstrated that ash had higher phosphorus concentrations at higher combustion temperatures [40]. Therefore, despite almost all the above-ground vegetation being consumed (basal area loss of 75–100% for approx. 75% of the Thomas Fire area) [44], the low to moderate soil burn severity resulting from the Thomas Fire [9] may suggest that fire intensity and temperature were relatively low, and this may have contributed to the lack of phosphorus leached from the ash. Furthermore, phosphorus has been shown to bind to large ash particles which are not transported far via the atmosphere [43,45,46] and therefore are not likely to contribute significantly to ash deposition outside the burn area. Although our data revealed that the Thomas Fire was not an important source of bioavailable phosphorus to the SBC, it is possible that differences in fire conditions, fuel type, and atmospheric transport may alter phosphorus dynamics during different wildfire events [40,43]. A complex combination of methodological (solubility differences in seawater, ash collection timing and location, creation of leachate, etc. [22,47]) and physical (atmospheric processing, soil–ash interactions, fuel type, fire conditions, etc.) factors probably determine the chemical composition of ash-seawater leachate and further study is urgently needed to determine how this variability may influence coastal ocean biogeochemistry.

Compared with other types of atmospheric aerosols, such as volcanic ash and desert dust, which can be important sources of iron and phosphorus [33,35,48], our results from the Thomas fire ash suggest that fires may be a more important nitrogen source to marine systems relative to other nutrients. While iron, phosphorus or other nutrients may sometimes limit primary production, many marine systems, including the California Current System, are considered nitrogen limited relative to average biological requirements of marine phytoplankton (N : P : Fe = 16 : 1 : 0.0075) [28,49,50]. Indeed, measurements of seawater nutrient concentrations showed sub micromolar nitrate concentrations and nutrient ratios indicated initial nitrogen deficiency relative to phosphorus and iron (N : P < 16 : 1 and N : Fe < 2133 : 1) [28] at the time of the Thomas Fire. The incubation experiments demonstrated that supplementary nitrogen provided by ash fuelled primary production with significant increases in POC concentrations while phosphate and nitrate + nitrite were depleted in ash-amended treatments (figure 3; electronic supplementary material, figure S1). This suggests that any additional nitrogen delivered to coastal marine waters at this time would enhance primary production. Although inorganic nitrogen concentrations were low and the deficiency relative to other nutrients suggest nitrogen limitation of microbial production, the addition of ash leachate-derived DOC, DON and micronutrients (e.g. iron) may have also contributed to the observed POC increase in ash treatments.

The annual timing of wildfires and ash deposition to the coastal ocean also probably impacts the response of these ecosystems. Inorganic nitrogen concentrations along the California coast display high seasonality with the highest concentrations in the SBC occurring during spring upwelling (March and April) and the lowest concentrations from July to November (average < 0.5 µM) [51]. Fire season in California typically coincides with times of the year with low overall nutrient concentrations in nearby coastal marine systems (summer through autumn). Wildfires in California may thus be important episodic sources of bioavailable nitrogen to nearby oceanic systems with the potential to relieve local nitrogen limitation and enhance primary production.

### (b) Estimated fertilizing effect of the Thomas Fire

Upwelling along the central and southern California coast is the largest source of nitrogen to coastal surface waters with estimated annual nitrogen fluxes of 7.5 × 10^8^ kg N yr^−1^ to the entire Southern California Bight [52] and 2.1 × 10^8^ kg N yr^−1^ to the SBC [53]. On smaller regional scales, atmospheric deposition and fluvial or wastewater discharge can be important sources of nitrogen [52]. For example, in the nearshore SBC surface waters, atmospheric nitrogen deposition from natural and anthropogenic sources was estimated to account for the largest annually integrated nitrogen flux (430 kg N km^−2^ yr^−1^) [52]. Spatio-temporal complexity of atmospheric aerosol deposition precludes precise quantification of Thomas Fire ash deposition to the SBC; however, based on the area burned [8], average above-ground live biomass (4.2 kg m^−2^) [54], and assuming 75–100% of vegetation was consumed with 50–80% of biomass undergoing complete combustion [40] we estimate that 0.7 × 10^9^–2.4 × 10^9^ kg of ash was produced by the Thomas Fire. If all ash produced was deposited throughout the SBC (approx. 100 km × approx. 40 km) [55], concentrations of ash in a 20 m deep mixed layer would average 0.01–0.03 g ash l^−1^. This range of values is slightly lower but comparable to the ash concentrations used here (0.08 g ash l^−1^ in the L treatment). Therefore, our treatments are probably relevant to conditions experienced during high deposition periods and may represent different surface ocean locations along a distance gradient from the fire source.

In total, leaching of nitrogen from the maximum estimated amount of ash based on our results (15 µM inorganic N g_(ash)_^−1^; figure 3*a*; electronic supplementary material, table S1–S2) would have added approximately 5 × 10^5^ kg N or approximately 126 kg N km^−2^ (29% of the annually integrated atmospheric flux) [52] to the SBC surface ocean during the 40 days of the Thomas Fire. Although this value is only 0.2% and 0.07% of the estimated nitrogen delivery to the SBC and the Southern California Bight via upwelling respectively, the Thomas Fire occurred during low nutrient (non-upwelling) conditions, representing an additional nutrient pulse that probably enhanced productivity. Assuming Redfield stoichiometry, this amount of nitrogen addition could stimulate up to approximately 2.9 × 10^6^ kg C of new production which is equivalent to approximately 3.5–14 times the estimated new production from large (not post-fire) river discharge events in the SBC [53]. Based on these estimates, dry ash deposition from large coastal wildfires is expected to fertilize adjacent marine ecosystems, especially during periods of low nutrient concentrations in surface waters, such as in the absence of upwelling.

Wet deposition and runoff during or after precipitation events would further increase wildfire-derived nitrogen (and other nutrient) loads to marine environments (figure 2). Measurements from burned watersheds feeding into the SBC have significantly increased concentrations of ammonium, nitrate and DON compared with unburned watersheds, especially during the first few storm events [12]. Wet deposition nutrient fluxes have also been estimated to be significantly higher than dry deposition fluxes during hazy, smokey conditions [17] indicating that rain can increase the rate of removal of nutrients from smoke and ash in the atmosphere and provide relatively larger nutrient pulses. These nutrient contributions would be temporally separated from dry deposition events because they depend on the timing of the first precipitation event after a wildfire. Rain also extinguishes wildfires, preventing further ash and smoke production. In the case of the Thomas Fire, a large precipitation episode occurred on 9 January 2018, before the fire was fully contained, resulting in a large debris flow event and increased fluvial input into the SBC [9]. Kelly *et al*. [15] reported increased concentrations of metals in river water during this rain event, presumably due to mobilization of fire derived materials, but suggested that the total amount of metals delivered to SBC waters due to fluvial transport was less than atmospheric deposition [15]. These additional processes impacting delivery of fire derived materials to coastal marine systems are probably significant and warrant further study.

### (c) Mechanism of ash fertilization; food for all?

The observed increase in total POC concentrations associated with increased ash concentrations indicates that ash leachate had an overall fertilizing effect on microbial communities. Interestingly, no single taxonomic group consistently grew to dominate the ash treatments. Rather, ASVs from many different taxonomic divisions displayed increased RA in ash-amended treatments and at a broad taxonomic level, the RA of most groups did not differ from control treatments despite the large increase in POC in ash-amended treatments. Notably, several small phytoplankton displayed higher RA in ash treatments compared with controls, potentially suggesting that small, fast-growing, autotrophs had an advantage over other protists. Although, more broadly, the RAs of primarily phototrophic taxonomic groups (e.g. Ochrophyta, Chlorophyta and Haptophyta) were not consistently higher in ash treatments compared with controls. Phytoplankton growth may also have been affected by enhanced grazing activity in response to the ash-fuelled increase in primary and/or bacterial production as the RA (and POC-scaled RA) of putative micrograzers (e.g. radiolarians and ciliates) typically did not significantly differ between ash and control treatments. The lack of a consistent taxonomic group displaying increased RA in response to ash exposure and the associated POC increase was unexpected given that diatoms (class Bacillariophyta) are often the dominant responders to nutrient enrichment with high growth rates [56,57] and form blooms following upwelling events off the coast of California [58,59]. Based on our observations, we hypothesize that pulsed deposition of wildfire ash acts as a ‘food for all’ in coastal marine ecosystems via leaching of a diverse mixture of inorganic nitrogen species (nitrate, nitrite and ammonium), silicic acid, metals and organic compounds.

The leaching of various chemical forms of nitrogen may have promoted the growth of multiple protistan taxonomic groups simultaneously since nitrogen metabolism within and across taxonomic groups varies based on the available nitrogen source [60–62]. For example, diatoms are often considered nitrate opportunists with several physiological and metabolic traits that allow for rapid nitrate uptake, assimilation and storage [62], while other groups (e.g. chlorophytes, cyanobacteria, dinoflagellates) are associated with more reduced forms of nitrogen [61,62] and become proportionally more abundant when ammonium rather than nitrate is supplied to natural communities [63]. Indeed, nitrate dominates the nitrogen supply delivered via upwelling [52] and usually promotes the growth of diatoms over other protist groups. Further, the magnitude of nutrient addition by wildfire ash deposition is small relative to nutrient delivery via upwelling events in the California Current. These short-term, small pulses of nutrients may also help explain the lack of a dominant ash-responder as favorable conditions may not persist long enough to maintain high growth rates of a particular group. Even with relatively small nutrient additions, some aerosol deposition studies using natural polluted aerosols, volcanic ash or desert dust report community shifts towards diatom dominance [64–67], though community responses are highly variable [32,64,68,69]. The response we observed after wildfire ash addition is thus probably driven by a combination of the various chemical forms of nitrogen and other nutrients leached by wildfire ash in addition to the magnitude of nutrient addition. It is also important to note that we only observed short-term responses to a single ash addition, so continuous pulses and longer-term impacts on protist communities are unclear.

### (d) No evidence of toxicity of wildfire ash

For the microbial community as a whole, it appears that any negative or toxic effects on protists were outweighed by the fertilizing effects of the ash leachate. Even though dinoflagellate RA was consistently decreased in ash treatments compared with controls by the end of both experiments, the estimated POC of Dinophyceae and Syndiniales suggests that, as a group, dinoflagellate growth was not inhibited by ash. It is, however, possible that toxic effects are present for some dinoflagellate species as species-specific toxicity responses have been reported for various phytoplankton [70]. Many Dinophyceae species exhibit slower growth rates than similarly sized cells from other taxonomic groups [56,71,72], potentially explaining their decreased RA without a concomitant decrease in POC in ash treatments. The class Syndiniales includes understudied putative marine parasites that appear to be ubiquitous and abundant in many marine systems [73,74]. Counterintuitively, we found that the RA of Syndiniales ASVs decreased in treatments with increased overall POC and (presumably) greater host density and a higher probability of host encounters. However, positive (possibly parasitic) associations of Syndiniales ASVs with Dinophyceae ASVs have been shown to be more common than with other protist groups [74], suggesting that Syndiniales primarily parasitize Dinophyceae species. Therefore, the reduction in host (Dinophyceae) RA may explain the reduced RA of Syndiniales ASVs.

Although at the community and broad taxonomic group levels there is generally a positive impact of ash addition on protist growth, it is possible that individual organisms within the community are more sensitive to pollutants released from the ash [22,23]. Paytan *et al*. [23] demonstrated toxic effects of atmospheric aerosols on marine microbial communities and attributed the toxicity to high concentrations of copper [23]. Differential sensitivities to copper were observed between picoeukaryotes, *Synechococcus* and *Prochlorococcus* while it has been suggested that larger cells are more resistant to copper toxicity [23,75]. We did not measure the growth or abundance of cyanobacteria, which may be highly sensitive to copper, while several small phytoplankton showed increased RA during ash exposure. Thus if toxic effects due to copper were present, it is not clear from these data. We found that copper leaches in high concentrations compared with other metals and is increased significantly in ash leachate (electronic supplementary material, table S3), but the toxicity of copper is controlled by the presence of Cu-binding organic ligands and the concomitant addition of DOC by ash may bind copper and prevent significant toxic effects [23,76]. Other toxic components of ash, such as polycyclic aromatic hydrocarbons (PAHs), may be affecting the growth of some species within these incubations but we did not measure PAH concentrations here and in previous work on wildfire ash, PAHs did not appear to correlate with toxicity [19,22]. Additionally, in studies on freshwater organisms that reported toxicity of wildfire ash or ash leachate, significantly higher concentrations (greater than 10×) [21,22] were used compared with those used here. These elevated concentrations are unlikely to be ecologically relevant when considering atmospheric ash deposition into a large ocean reservoir.

### (e) Conclusions and considerations for future research

Our findings suggest that large wildfires can be important sources of nitrogen and other nutrients to coastal marine ecosystems where they can fuel productivity and maintain a highly diverse protistan community assemblage. Near areas of active burning, wildfires are probably the dominant atmospheric source of nitrogen to coastal areas and under certain conditions, they could contribute significantly to total marine nitrogen inputs, a factor of increasing importance as fire frequency and severity rises in a changing climate. The trends observed here provide a basic understanding of how marine protist communities respond to wildfire ash while shedding light on the importance of an understudied mechanism of nutrient delivery to marine ecosystems.

Future research should focus on characterizing wildfire ash leaching in seawater across fire conditions, fuel sources and environments. This will allow for estimates of wildfire ash nutrient inputs at broader spatial scales with implications for global ocean biogeochemical cycling. It is also critical to measure and explore other processes influencing the delivery of wildfire-derived materials to marine environments, including atmospheric evolution and transport and terrestrial debris flows. In the context of biological consequences of wildfire impacts in the marine environment, we report only on short-term responses of microbial communities to ash leachate generated by a single wildfire in coastal CA, but longer-term impacts and the mechanisms controlling the biological responses should be further investigated. Tightly controlled experiments on individual chemical components of ash and different microbial or protist groups may help identify specific mechanisms of biological responses. Additional biological effects of wildfires and ash including reduced light availability due to smoke and ash cover and increased particle concentrations in surface waters should also be considered. Overall, links between wildfires and marine ecosystems should be a priority for future studies as global climate change is significantly altering wildfire dynamics and the implications for marine systems are currently unknown.

## Data Availability

The 18S metabarcoding sequence data and ancillary data are deposited in the Sequence Read Archive (National Center for Biotechnology Information) under accession number PRJNA719165. The data are provided in electronic supplementary material [77].
